# Retraction: The influence of gradient and porous configurations on the microwave absorbing performance of multilayered graphene/thermoplastic polyurethane composite foams

**DOI:** 10.1039/d1ra90102h

**Published:** 2021-04-16

**Authors:** Chaozhi Wang, Jiang Li, Shaoyun Guo

**Affiliations:** The State Key Laboratory of Polymer Materials Engineering, Polymer Research Institute of Sichuan University Chengdu 610065 China li_jiang@scu.edu.cn

## Abstract

Retraction of ‘The influence of gradient and porous configurations on the microwave absorbing performance of multilayered graphene/thermoplastic polyurethane composite foams’ by Chaozhi Wang *et al.*, *RSC Adv.*, 2019, **9**, 21859–21872, DOI: 10.1039/C9RA04735B.

We, the named authors, hereby wholly retract this *RSC Advances* article due to concerns affecting the reliability of the data.

Fig. 3d is exactly the same as Fig 3d in our related paper “A versatile strategy for alternately arranging the foam ratio layers of multilayer graphene/thermoplastic polyurethane composite foams towards lightweight and broadband electromagnetic wave absorption” (Chaozhi Wang *et al.*, *RSC Adv.*, 2019, **9**, 23843–23855), however, the experimental procedures of these two papers are different, with different foam ratio and graphene content, so the same picture cannot be used to illustrate different samples.

In Fig 8, (a)–(d) represent 2–5 layers of TPU/graphene multilayer samples, respectively. However, all the images are taken from a picture of the same electron microscope image under different magnification rates, which cannot prove the microscopic morphology of the samples with different layers. At the same time, the size labeling is incorrect. The magnification ratios of (a)–(d) are different, so the size labeling cannot be all 3 mm.

On page 21864 of the article, it states “The graphene, selectively dispersed over the cellular walls by foaming, formed a three-dimensional conductive network structure, as shown in the Fig. 6b”. However, in Fig. 6b, the three-dimensional conductive network structure and graphene’s selective dispersion and alignment in the pore structures cannot be seen very clearly, because it is only a cell structure diagram, and therefore does not prove the existence of a three-dimensional conductive path.

As the above data errors and misuse of pictures have greatly influenced the reliability of the conclusions, we are retracting this article to protect the integrity and accuracy of the scientific record.

Signed: Chaozhi Wang, Jiang Li and Shaoyun Guo

Date: 5^th^ April 2021

Retraction endorsed by Laura Fisher, Executive Editor, *RSC Advances*

## Supplementary Material

